# What the Heck?—Automated Regioselectivity Calculations of Palladium-Catalyzed Heck Reactions Using Quantum Chemistry

**DOI:** 10.1021/acsomega.2c06378

**Published:** 2022-12-02

**Authors:** Nicolai Ree, Andreas H. Göller, Jan H. Jensen

**Affiliations:** † Department of Chemistry, University of Copenhagen, Universitetsparken 5, 2100 Copenhagen Ø, Denmark; ‡ Bayer AG, Pharmaceuticals, R&D, Computational Molecular Design, 42096 Wuppertal, Germany

## Abstract

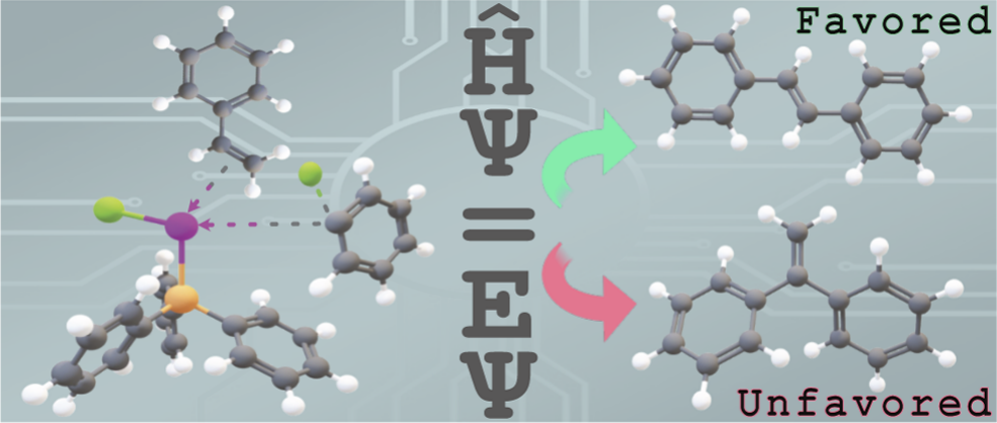

We present a quantum chemistry (QM)-based method that computes the relative energies of intermediates in the Heck reaction that relate to the regioselective reaction outcome: branched (α), linear (β), or a mix of the two. The calculations are done for two different reaction pathways (neutral and cationic) and are based on *r*^2^SCAN-3c single-point calculations on GFN2-xTB geometries that, in turn, derive from a GFNFF-xTB conformational search. The method is completely automated and is sufficiently efficient to allow for the calculation of thousands of reaction outcomes. The method can mostly reproduce systematic experimental studies where the ratios of regioisomers are carefully determined. For a larger dataset extracted from Reaxys, the results are somewhat worse with accuracies of 63% for β-selectivity using the neutral pathway and 29% for α-selectivity using the cationic pathway. Our analysis of the dataset suggests that only the major or desired regioisomer is reported in the literature in many cases, which makes accurate comparisons difficult. The code is freely available on GitHub under the MIT open-source license: https://github.com/jensengroup/HeckQM.

## 1 Introduction

**Figure 1:**
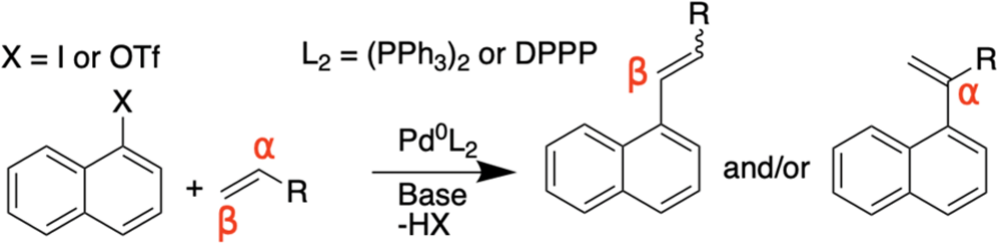
Example of intermolecular Heck reactions. The combined use of triflate (OTf) and bidentate Pd ligands (DPPP) tends to favor the α regioisomer (cf. Tables 1 and 3).

The Heck reaction has become an indispensable tool for the formation of aryl (or vinyl)-alkene C–C bonds that often might otherwise be difficult to assemble.^1,2^ The Heck reaction can give rise to linear (β) and branched (α) isomers (Figure 1) where the regioselectivity for intermolecular Heck reactions is governed by the catalyst, reagents, and the nature of aryl and alkene substituents X and R. The rule of thumb for the effect of R on the regioselectivity is that electron-withdrawing groups (EWGs) tend to give the β isomer while electron-donating groups (EDGs) tend to give mixtures. Certain combinations of X, R, catalyst, and reagents tend to favor the α isomer, by favoring a different (“cationic”) reaction pathway than the more conventional (“neutral”) pathway. However, there are many exceptions to these rules as we discuss below and some have even argued that “we cannot make any predictions about the regiochemistry of arylation ··· and temptations to find simple rules should be discarded.”^3^ There is thus a great deal of interest in gaining a better understanding of the factors that govern the regioselectivity both theoretically^4−6^ and experimentally.^3^

On the theoretical side, Deeth et al.,^4^ Bäcktorp and Norrby,^5^ and Domzalska-Pieczykolan et al.^6^ have explained experimentally observed regioselectivity based on density functional theory (DFT)-computed barrier heights of the insertion step. Deeth et al. have developed a predictive model based on DFT-computed electrostatic and orbital properties of the neutral or cationic alkene–catalyst complexes, which they test on eight different alkenes. They use a very simplified model of the Pd catalyst (PdIPH_3_ and Pd(PH_3_)_2_ for the neutral and cationic pathway, respectively), and the aryl group is replaced by ethene, but still they get a good agreement with the experimentally observed regioselectivities.

**Figure 2:**
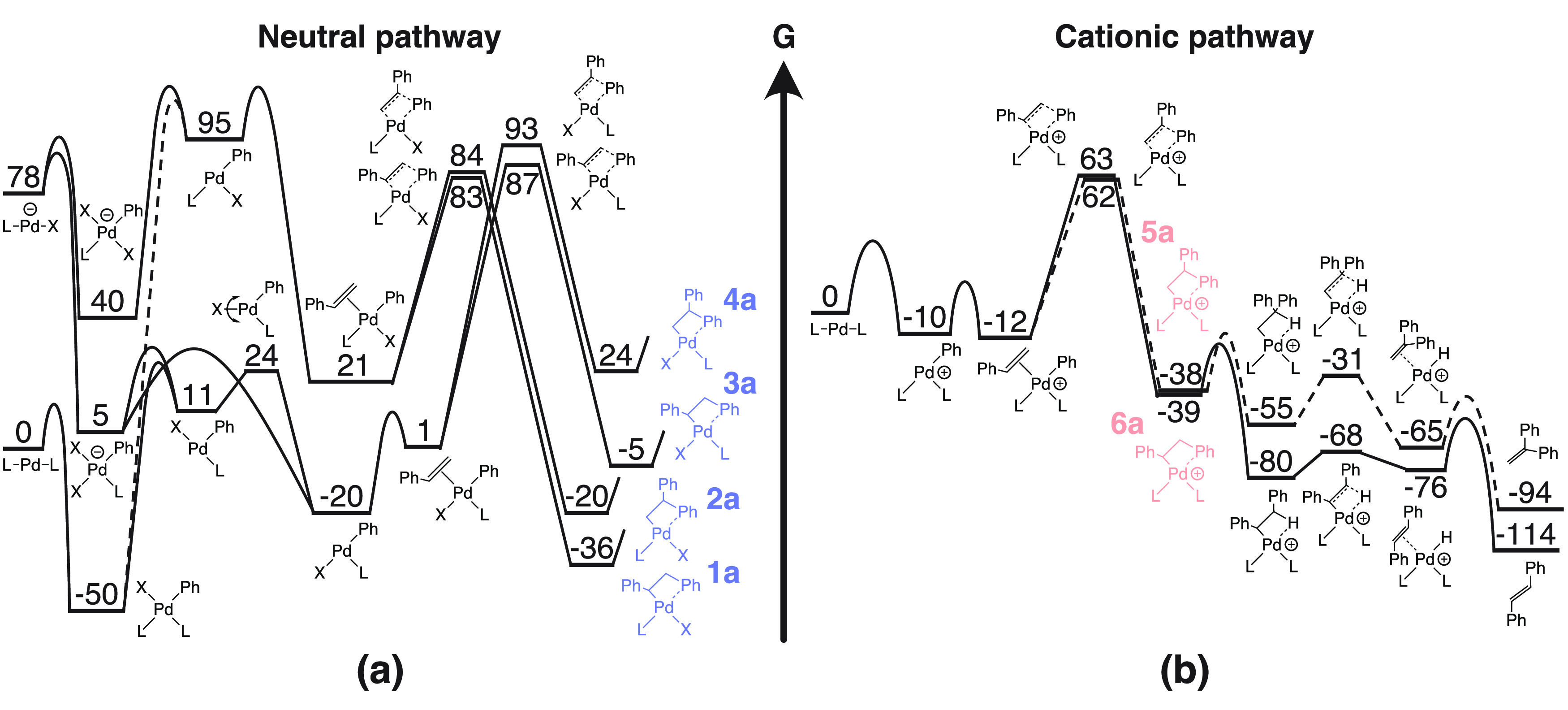
Heck reaction of styrene and phenyl chloride in terms of (a) the neutral pathway and (b) the cationic pathway with free energies in kJ/mol adapted from the work of Bäcktorp and Norrby.^5^ The highlighted structures (**1a**–**6a**) are the post-insertion complexes that form the basis for the regioselectivity calculations.

Bäcktorp and Norrby investigated the entire reaction paths for both the neutral and cationic mechanisms of styrene and phenyl chloride catalyzed by Pd(PPh_3_)_2_, including the two neutral paths depending on whether the chloride is *cis* or *trans* to the phenyl ring (Ph) when complexed with the catalyst (Figure 2). The computed barrier heights for the insertion steps of the neutral path (where the chloride is *trans* to Ph) and the cationic path match the experimentally observed regioselectivities of predominately β and a roughly equal mix of α and β, respectively. Their results suggest that under some relatively common reaction conditions, both reaction mechanisms may contribute to the regioselectivity. As the authors note, the relative importance of each path can, in principle, be computed using DFT, but the difference in charge makes the results very sensitive to the solvent model, and it is not clear whether the accuracy is sufficient to be used in practice.

While DFT-computed barrier heights appear to be able to predict the regioselectivities, this has really only been tested for a handful of systems. The reason is that it is difficult to automate the location of transition states (TSs) in a reliable manner. We noted that the relative energies of the post-insertion complexes, computed by Bäcktorp and Norrby, correlate reasonably well with the respective relative barrier heights (Figure 2). These relative energies are much easier to compute in an automated manner, and we decided to test whether this correlation holds in general. We present a fully automated QM-based workflow for determining the regioselectivity of intermolecular Heck reactions that is based on the calculated relative energies of these post-insertion complexes following the carbopalladation step. The method applies to cross-couplings of monosubstituted alkenes with aryl/vinyl halides (Cl, Br, or I) or triflates in the presence of a base and a palladium (Pd) catalyst with monodentate triphenylphosphine (PPh_3_) ligands to form branched (α) and/or linear (β) disubstituted alkenes. In fact, the workflow can handle intramolecular Heck reactions, but we will focus on the intermolecular Heck reactions and test the approach on several thousands of such reaction outcomes.

## 2 Methods

### 2.1 Automated Quantum Chemistry

**Figure 3:**
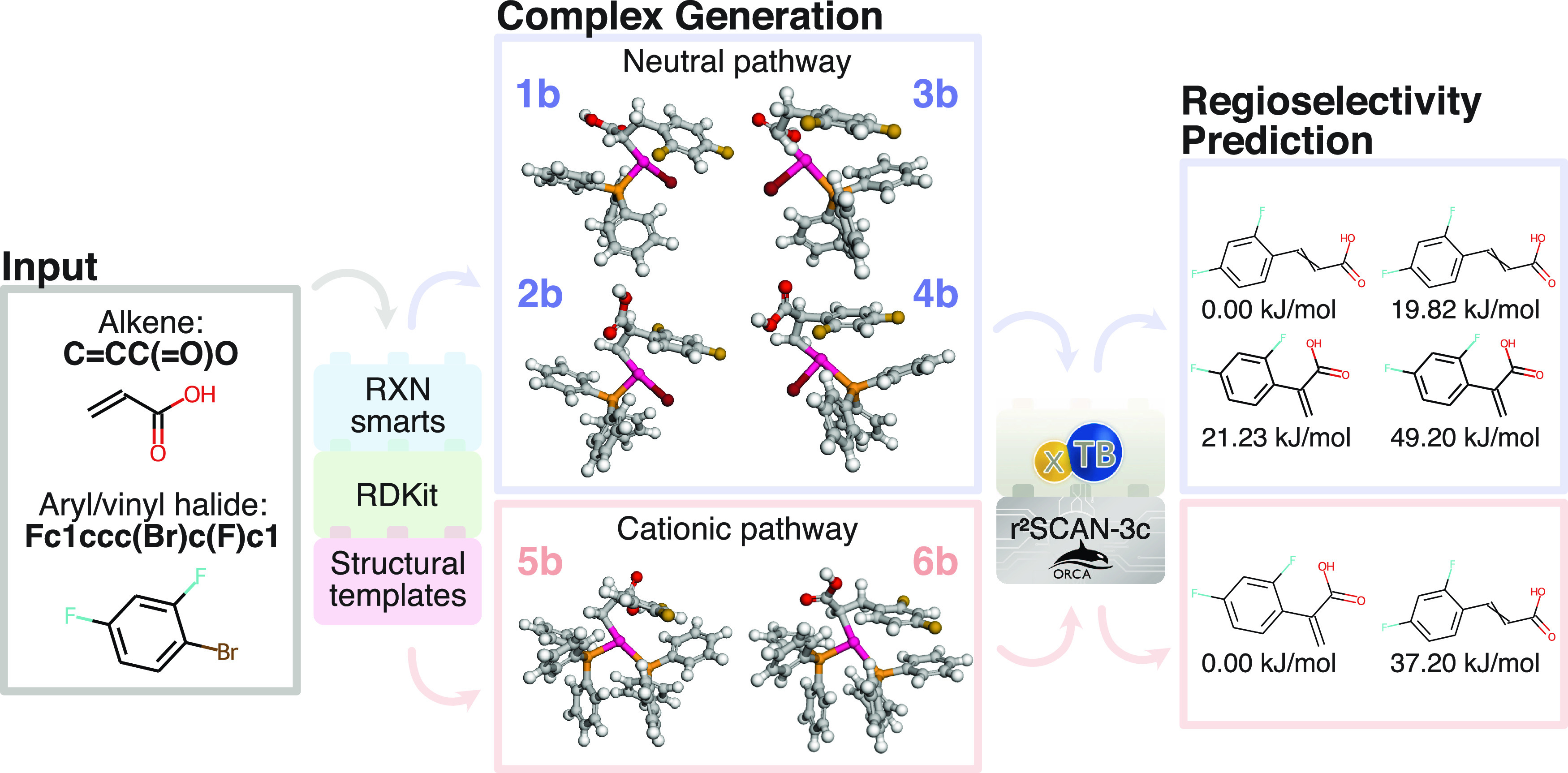
Schematic description of the automated workflow for determining the regioselectivity of intermolecular Heck reactions (see Figure S1 in the Supporting Information for a detailed flowchart of the procedure). Note that the *cis*/*trans* stereochemistry of the β-products is not specified as this is determined later in the reaction mechanism (see Figure 2b). However, the *trans* isomer is more prevalent among the intermolecular β-products in the Reaxys dataset. The example reaction has the following Reaxys ID: 4892001.

An overview of the automated workflow can be seen in Figure 3. Starting from simplified molecular input line entry system (SMILES) representations of a monosubstituted alkene, RC=C, and an aryl or vinyl halide/triflate, R–X, the workflow automatically generates all possible post-insertion complexes for both the neutral and cationic pathway (compounds **1**–**6** in Figures 2 and 3). This is done by applying a set of reactions SMARTS in combination with the RunReactants function in RDKit.^7^ We assume the usual order of reactivity for X: I ≫ Triflate > Br ≫ Cl, so that, for example, if an aryl group has both I and Br substituents, the reaction is assumed to occur at the I site. If there are multiple sites with the most reactive halide, then reactions at all sites are investigated (see Figures S9–S13). For each post-insertion complex, min(1 + 3·*n*_rot_, 80) conformers are generated, where *n*_rot_ is the number of rotatable bonds of the products, using constrained embedding with respect to modified structural templates retrieved from the mechanistic study by Bäcktorp and Norrby.^5^ The modified structural templates consist of the Pd catalyst (with Cl replaced by the reacting halide/triflate if not Cl for the neutral pathway) and a few essential atoms for connecting the reactant(s) to the catalyst (graphical representations of the structural templates can be seen in Figure S2 in the Supporting Information). Only spatially unique conformers are carried forward by selecting the centroids from a Butina clustering with RDKit using a pairwise heavy-atom position root-mean-square deviation (RMSD) with a threshold of 0.5 Å. The conformers are then prescreened with constrained force-field optimizations in phenol (PhOH, dielectric constant = 12.4) using GFNFF-xTB^8^ and the analytical linearized Poisson–Boltzmann (ALPB) solvation model^9^ as implemented in the open-source semiempirical software package xtb.^10^ The constrained atoms are automatically identified by applying a substructure match between the post-insertion complex and the corresponding structural template. Following the prescreening, conformers with total energies greater than 10 kJ/mol of the lowest-energy conformer are removed and the remainder are filtered using the Butina clustering algorithm to extract only unique conformers. The constrained optimization procedure is now repeated by replacing the force field with the semiempirical tight-binding GFN2-xTB model,^11^ and followed by a full relaxation of the remaining conformers at the same level of theory. After all optimizations, the input and output structures are compared by converting the Cartesian coordinate file (.xyz) into a structure-data file (.sdf) using Open Babel 3.1.0.^12^ If the atom connectivity (except for bonds to Pd) is different due to a broken/created bond, the energy of the conformer is set to 60,000 kJ/mol. Hence, such conformers are removed as a consequence of the energy cutoff. For more accurate energies of the post-insertion complexes, single-point density functional theory (DFT) calculations are performed in dichloromethane (DCM, dielectric constant = 9.08) using the *r*^2^SCAN-3c composite electronic structure method^13^ and the conductor-like polarizable continuum model^14,15^ (C-PCM) as implemented in the quantum chemistry program ORCA version 5.0.1.^16^ A graphical output is then presented to the user for each pathway, which shows the potential products in order of increasing energies of the post-insertion complexes.

### 2.2 Reaction Dataset

We have conducted a careful curation of literature data retrieved from the Reaxys repository and used it in the design and testing of the automated workflow for determining the regioselectivity of intermolecular Heck reactions. The reaction data are limited to cross-couplings of monosubstituted alkenes with unsaturated halides (or triflates) in the presence of a base and a Pd catalyst. In an attempt to avoid additives that may alter the regioselectivity, we focus on commonly employed precatalysts for obtaining the desired and active Pd(0)-catalyst and excluded reactions using lithium compounds (lists of precatalysts and included/excluded additives are provided in the Supporting Information).

The data curation mainly consists of a template approach, where a set of reaction SMARTS are used to generate all possible products from detected coupling partners. A reaction is then classified as being correct, if a reported product in the reaction SMILES from Reaxys matches one of the generated products. However, only reactions leading to a product with a molecular weight (*M*_W_) of ≤600 g/mol are considered (average *M*_W_ = 334 g/mol), and reaction duplicates are removed. The template approach allows us to divide the reactions into different categories such as inter- or intramolecular reactions leading to either α or β products or a mix.

The dataset is available via a single Reaxys query and a provided list of Reaction IDs separated by semicolons. Further instructions for retrieving and cleaning the data are provided in the associated GitHub repository.

## 3 Results and Discussion

### 3.1 Comparison to Bäckstrom and Norrby

Bäckstrom and Norrby predict an energy difference between 4a and 3a of 29 kJ/mol (Figure 2), with 3a being the lowest, while our methodology predicts an energy difference of 52 kJ/mol. Similarly, the energy difference between 2a-1a and 5a-6a is 16 and 1 kJ/mol, respectively, while our model predicts 27 and 5 kJ/mol. Thus, while our model predicts the correct isomer or mixture in all three cases, it overestimates the energy difference between the two isomers. Note that the difference in the barriers is significantly smaller than the energy difference of the intermediates for the neutral pathways. It is thus difficult to predict mixtures for neutral pathway based on the relative energies of intermediates. We use 3 kcal/mol ≈ 12.6 kJ/mol as a cutoff for the accuracy of our computational method, indicating that two isomers are roughly equal in energy according to our theoretical model. But larger energy differences could be computed for cases where mixtures are observed for the neutral pathway.

### 3.2 Cabri Dataset

In this section, we focus on a handful of reactions that are often used to exemplify the rules of thumb and test the computational calculations (Table 1). Most of the experimental data has been obtained by Cabri and co-worker^17^ for the reactions shown in Figure 1. Our methodology generally predicts the β selectivity for the neutral reaction pathway mechanism, except when the alkene substituent is 2-pyrrolidone where the energy difference between the two intermediates is less than 3 kcal/mol ≈ 12.6 kJ/mol. Thus, for the reasons discussed in the previous subsection, we generally are not able to predict the alkene substituents that give rise to a mixture of regioisomers under neutral conditions.

**Table 1:** Experimental and Predicted Regioselectivities for the Reactions Shown in Figure 1^a^

			neutral		cationic	
			exp α/β	prediction	exp α/β	prediction
a	*O*-*n*-butane	EDG	76/24	β	>99/1	β
b	2-pyrrolidone	EDG	64/36	m	>99/1	α
c	N(CH_3_)COCH_3_	EDG	71/29	β	99/1	α
g	OAc	EDG	65/35	β	95/5	β
i	*N*-succinimide	EDG	5/95	β	62/38	α
f	CH_2_CH_2_OH	EDG	20/80*	β	90/10*	α
	*n*-butane	EDG	20/80*	β	80/20*	m
h	Ph	EWG	7/93	β	38/62	m
d	CH_2_OH	EWG	0/100*	β	>99/1	α
e	CH(OH)CH_3_	EWG	10/90*	β	95/5	α
k	cyano	EWG	<1/99	β	<1/99	β
j	COOCH_3_	EWG	<1/99	β	<1/99	β

a The experimental values are from refs (17, 18) (the latter is indicated by a*). The neutral results are obtained with monodentate (PPh_3_)_2_ Pd ligands and X = I, while the cationic results are obtained for X = triflate and the bidentate DPPP ligand. α, β, and “m” indicate the predicted selectivity or a mix where the two isomers are within 3 kcal/mol ≈ 12.6 kJ/mol. EDG and EWG indicate whether the alkene R-group is electron-donating and -withdrawing, respectively. The letters in the leftmost side indicate the label used in ref (17). As discussed in the text, our method is not expected to be able to predict all cases where a mix should be observed for the neutral pathway.

For the cationic pathway, our method fails to predict α selectivity for butoxy (*O*-*n*-butane) and acetyloxy (OAc) and fails to predict a mix for *N*-succinimide. Since we are doing a relatively limited conformational search, we have tested whether the calculations improve by performing a systematic search with up to 50,000 conformers (Table S1) or a molecular dynamics (MD)-based search (Table S2), but the results for these substituents do not change. In fact, a more thorough conformational search in general leads to slightly worse agreement with the experiment. The better results of the more limited conformational search are most likely due to a better error cancellation since the ligand conformations are more similar in the two intermediates.

Another possible source of error is the use of *r*^2^SCAN-3c, which employs a relatively small basis set. We thus compared the *r*^2^SCAN-3c results to corresponding B3LYP/def2-TZVP and DLPNO-CCSD(T)/def2-TZVPP results (Figures S7 and S8) for the first entry in Table 1, but all three methods lead to a prediction of β selectivity. So neither conformational sampling nor QM level of theory can explain the few discrepancies with the experiment observed for this dataset.

However, overall our method is able to predict the switch from β to α selectivity, or the lack thereof for cyano and methoxycarbonyl (COOCH_3_), when going from neutral to cationic reaction conditions.

### 3.3 Full Reaction Dataset

#### 3.3.1 Analysis of the Reaxys Dataset

Here, we investigate to what extent the usual rules of thumb for selectivity are reflected in the reported selectivities in the literature. We found 14,240 and 663 inter- and intramolecular Pd-catalyzed Heck reactions in Reaxys, using the search criteria described in the Supporting Information. Out of the 14,240 intermolecular reactions, 628 and 13,041 give either α or β selectivity, respectively, while 571 give a mix of α and β. However, it should be kept in mind that studies sometimes only report the sought-after isomer. For example, we found two pairs of studies that report opposite selectivities for identical reactants and reaction conditions (Figure S3). Bidentate Pd ligands, along with either nonhalogen aryl substituents or halide scavengers, are usually taken as a prerequisite for the cationic path. However, in many cases, only the catalyst precursor, e.g., palladium(II) acetate (Pd(OAc)_2_), is listed in Reaxys which do not necessarily mean that the reactions are carried out without Pd ligands. We therefore focus on reactions that explicitly use a Pd catalyst with canonical monodentate (PPh_3_) or bidentate (DPPP or DPPE) ligands, which are also the single most abundant ligands for reactions with α and β selectivities, respectively. This leaves us with 287 and 2988 reactions with α and β selectivity, where the vast majority of ligands are bi- and monodentate, respectively. In addition, there are 67 reactions that result in a mix of isomers, of which 46 use bidentate Pd ligands. Next, we determined the number of reactions that were performed using either triflate alkyl ligands or halide scavengers (Table 2). These results show that out of the 287 reactions that are reported as α-selective, only 99 (34%) were obtained with the reaction conditions that are generally thought necessary to produce α selectivity.

**Table 2:** Overview of the Regioselectivity of 14,240 Intermolecular Heck Reactions Found in the Reaxys Database^a^

	α	β	mix	total
total	628	13,041	571	14,240
Pd(Ac)_2_	181	6473	163	6817
DPPP or DPPE	268	116	46	430
(PPh_3_)_2_	19	2872	21	2912
cationic conditions	99	42	13	154
R = [O, N] or C[O, N]	260	211	19	490
R = C=O or Ar	9	2246	16	2271

a There are 6817 reactions where the Pd ligands are not defined, 430 + 2912 = 3342 reactions using DPPP, DPPE, or (PPh_3_)_2_ ligands. Of those, 154 reactions are performed under “cationic” conditions or have similar groups of alkene ligands (see text).

To sum up, the main difference for α vs β selectivity is the much higher percentage of bidentate ligands (93 vs 4%, respectively) when α is observed, although early work by Cabri suggests that this alone does not result in α selectivity (Table 3). Another difference between the α and β set is the alkene substituent. For the α set, in 91% of the cases, the atom bonded to the alkene is either O or N or an aliphatic C bonded to O or N, while for the β set, 75% of the corresponding atoms are either a carbonyl (mostly esters) or an aromatic carbon (Table 2). So, bidentate ligands (either DPPP or DPPE) plus ED alkene substituents involving N or O account for 254 of the 287 reactions where α selectivity is observed. Similarly, monodentate ligands ((PPh_3_)_2_) plus EW alkene substituents involving either carbonyls or aromatic groups account for 2154 of the 2988 reactions where β selectivity is observed.

**Table 3:** Some of the Experimentally Measured Ratios of Regioisomers (α/β) That Support Two Alternative Pathways^19^^a^

X	(PPh_3_)_2_	DPPP	DPPP + Ag^+^/Tl^+^
Br	76/24^b^	61/39	>99/1
OTf	63/37	>99/1	

a The reaction is shown in Figure 1 for different additives, Pd ligands, and X.

b I is used in instead of Br.

#### 3.3.2 Results of Calculations

We now apply our QM methodology to cases employing a Pd catalyst with one of the following ligands: DPPP, DPPE, or (PPh_3_)_2_ for both the neutral and cationic pathways. We remove 31 reactions due to convergence problems and an additional 10 that have several reaction sites (we discuss these separately below). These 41 reactions all produce the β isomer experimentally, leaving us with 2947 such cases. Using the neutral pathway, we predict the α and β isomers for 7 and 64% of the reactions and a mix for the remaining 29%, while the corresponding percentages for the cationic pathway are 32, 37, and 31%. Thus, our method predicts that the intermediates associated with the α and β isomers are very similar in energy (<3 kcal/mol ≈ 12.6 kJ/mol) for close to a third of the reactions, while a mixture is reported for only 2% of the cases. In 26% of the cases, both pathways agree on a regioisomer or a mix, and these calculations agree with experiment in 3, 88, and 3% of the cases for α, β, and mix, respectively. Thus, if both pathways predict the β isomer, then there is a good chance that this will be observed experimentally; however, that is not the case for the α isomer or a mix.

**Figure 4:**
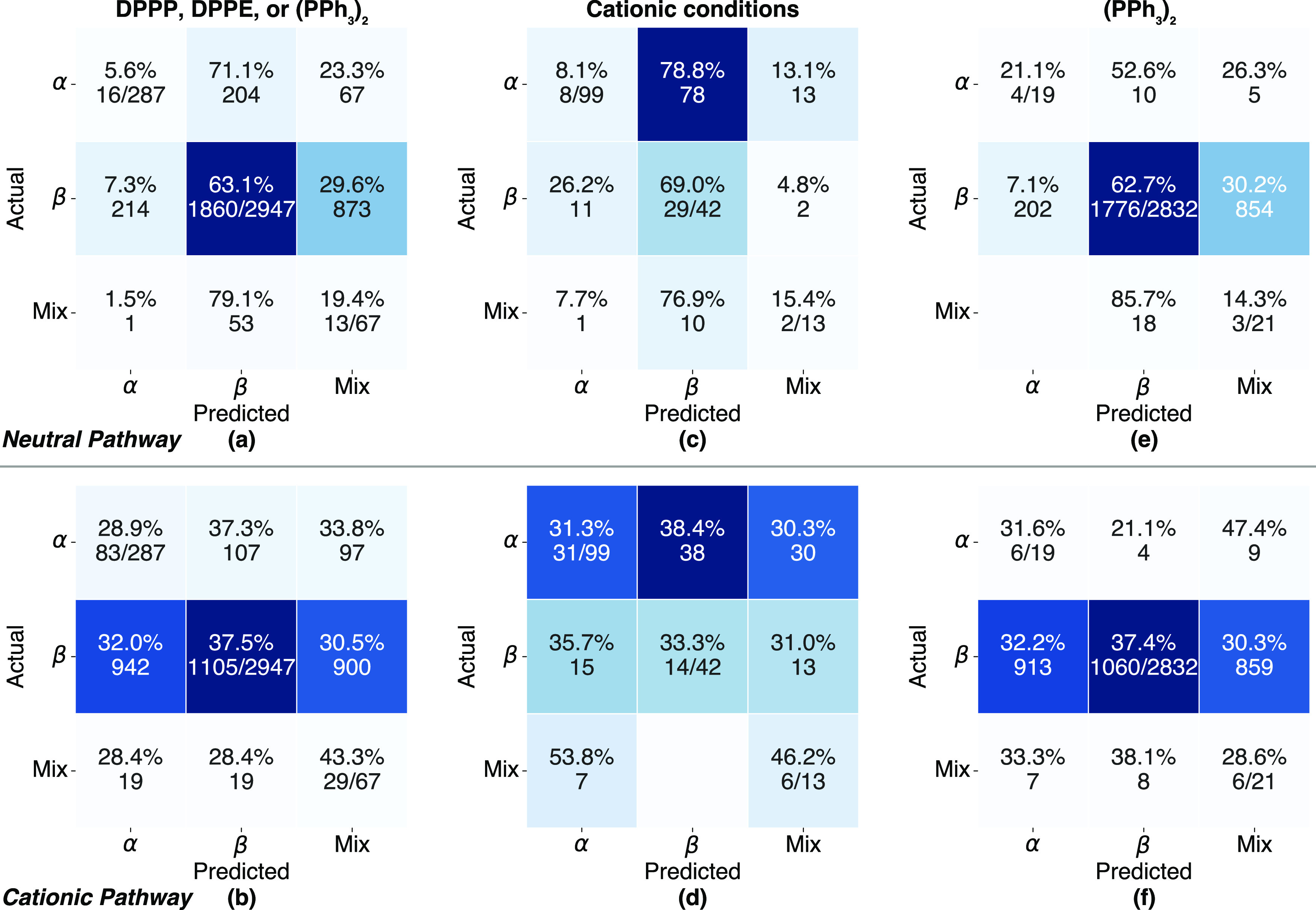
Confusion matrices for the QM calculations of the reactions with DPPP, DPPE, or (PPh_3_)_2_ Pd ligands (a, b), run under cationic conditions (c, d), or with (PPh_3_)_2_ ligands (e, f). Calculations for the neutral and cationic pathways are on the top and bottom, respectively.

Overall, the neutral path is the best predictor of β isomers, with an accuracy of 63%. However, this path also leads to 204 β predictions that are in fact α. The cationic path is a better predictor of α and mix, but with accuracies of only 29 and 43%. The accuracy of the cationic path calculations does not improve notably for the subset with 153 products that are obtained under cationic conditions as can be seen in Figure 4d (the large percentage increase in false mix predictions for the α isomer is likely due to the small numbers involved). Similarly, the accuracy is relatively unchanged in going to the monodentate subset (Figure 4e,f), keeping in mind that the relatively low number of observed α products and mix can make percentage changes unreliable. So the fact that our calculations are based on the (PPh_3_)_2_ ligand, while the majority of the experimental data is obtained with the DPPP or DPPE bidentate ligands does not appear to be a source of the low predictive accuracies for α and mix.

The most likely source of discrepancy is the reporting of a mix of isomers in the literature, where it is not uncommon to only report the major or desired isomer. While there is no ideal way to deal with this issue, we can get a rough estimate of the effect by assigning the mix predictions to the observed regioisomer. For example, a mix is predicted for 873 reactions using the neutral pathway in cases where the reported outcome was β, and if this amount is added to the 1860 correct β predictions, the accuracy of this “not α” prediction increases to 93%. Similarly, the accuracy of the “not β” prediction for the cationic path increases to 63%.

Finally, we turn our attention to the 10 cases where there are two possible halide reaction sites (Figures S9–S13). Our methodology attempts to predict the most reactive site in addition to the regioselectivity by comparing the relative energies of all isomers. However, in 8 of the 10 cases, the neutral pathway predicts a mix of isomers including the experimentally observed one using the 3 kcal/mol ≈ 12.6 kJ/mol cutoff. For the remaining two cases (Figure S13), our method agrees with an experiment in one case but disagrees in another.

## 4 Conclusions and Outlook

We present a quantum chemistry (QM)-based method that computes the relative energies of intermediates in the Heck reaction that relate to the regioselective reaction outcome: branched (α), linear (β), or a mix of the two (Figure 1). The calculations are done for two different reaction pathways (neutral and cationic) and are based on *r*^2^SCAN-3c single-point calculations on GFN2-xTB geometries that, in turn, derive from a GFNFF-xTB conformational search. The method is completely automated and is sufficiently efficient to allow for the calculation on thousands of reaction outcomes.

The method can reproduce prior DFT results^5^ as well as a systematic experimental study where the ratios of regioisomers are carefully determined (Table 1), although there are a few discrepancies for the latter. These discrepancies are apparently not caused by insufficient conformational sampling or level of theory, and their exact cause remains unclear.

We assembled a larger dataset by searching Reaxys for Heck reactions where the Pd ligands resembled the one used in our QM methodology. Of these 3342 reactions, only 287 have α-selectivity, 67 are a mix of the two, while the rest have β-selectivity. Only 99 of the α-selective reactions were obtained under cationic reaction conditions (primarily bidentate Pd ligands and an aryl-triflate, cf. Table 3) generally thought necessary to achieve exclusive α-selectivity. Also, electron-donating groups (EDGs) on the alkene are generally thought to give mixtures or α-selectivity depending on the reaction conditions. However, 43% of reactions involving strong EDGs have reported β-selectivity. This, combined with the relatively low percentage (2%) of reported isomer mixtures, could suggest that only the major or desired regioisomer is reported in the literature in many cases.

Comparing the QM-based calculations to reaction outcomes reported in Reaxys, the results are somewhat worse with accuracies of 63% for β-selectivity using the neutral pathway and 29% for α-selectivity using the cationic pathway. The performance is not improved for reaction subsets where the reaction conditions are better established or the Pd ligand corresponds exactly to the one used in our QM methodology. Our method predicts regioisomer mixtures in about 30% of the cases and one likely source of discrepancy is that such mixtures are rarely reported in the literature as discussed above, or that the regiospecific outcome is optimized using reaction conditions that are not accounted for in our model. Furthermore, the difference between a 95/5 and 50/50 product ratio, where the former is clearly regioselective, is only a few kJ/mol—a level of accuracy that is difficult to obtain.

## Notes

The code for the automated workflow and Reaxys Reaction IDs along with code for data preparation are available at https://github.com/jensengroup/HeckQM.

## Supporting Information Available

Flowchart of the automated workflow; graphical representations of the structural templates; results of extending the conformational sampling or increasing the computational level of theory; dataset retrieved from the Reaxys repository; and results for reactions with multiple selectivity sites (PDF)
