# Supplementary material for: What the Heck?—Automated Regioselectivity Calculations of Palladium-Catalyzed Heck Reactions Using Quantum Chemistry
Source: ACS Omega. 2022 Dec 2;7(49):45617–23. doi: 10.1021/acsomega.2c06378 (PMC9753166; doi:10.1021/acsomega.2c06378)
Supplement: Supplementary file 1 — ao2c06378_si_001.pdf [file ao2c06378_si_001.pdf]

Supporting Information

What the Heck? – Automated regioselectivity calculations of palladium-catalyzed Heck reactions using quantum chemistry

Nicolai Ree,† Andreas H. Göller,*,‡ and Jan H. Jensen*,†

†*Department of Chemistry, University of Copenhagen, Universitetsparken 5, 2100 Copenhagen Ø, Denmark*

‡*Bayer AG, Pharmaceuticals, R&D, Computational Molecular Design, 42096 Wuppertal, Germany*

E-mail: andreas.goeller@bayer.com; jhjensen@chem.ku.dk

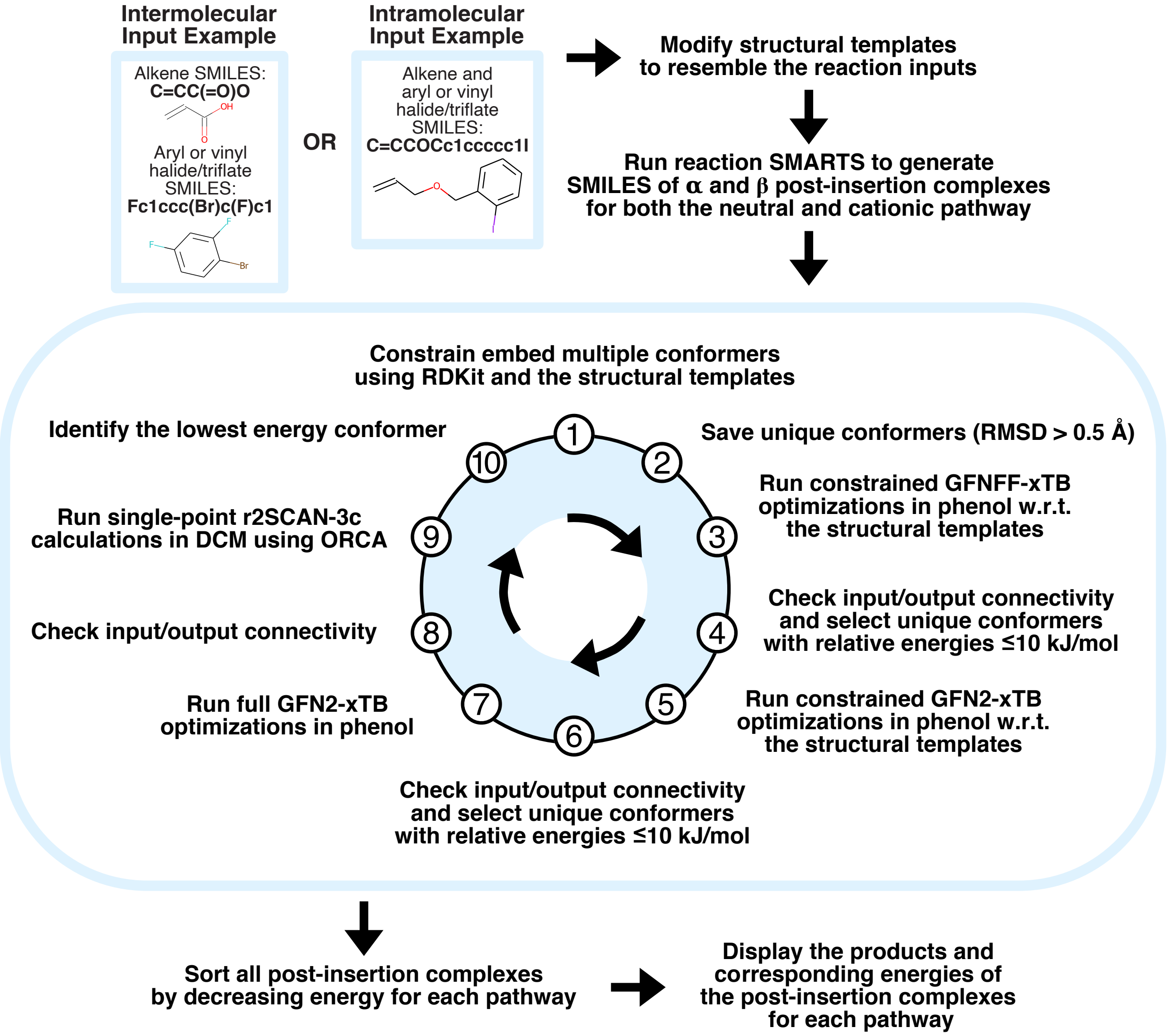

Figure S1: A detailed flowchart describing the automated workflow for determining the regioselectivity of palladium-catalyzed Heck reactions.

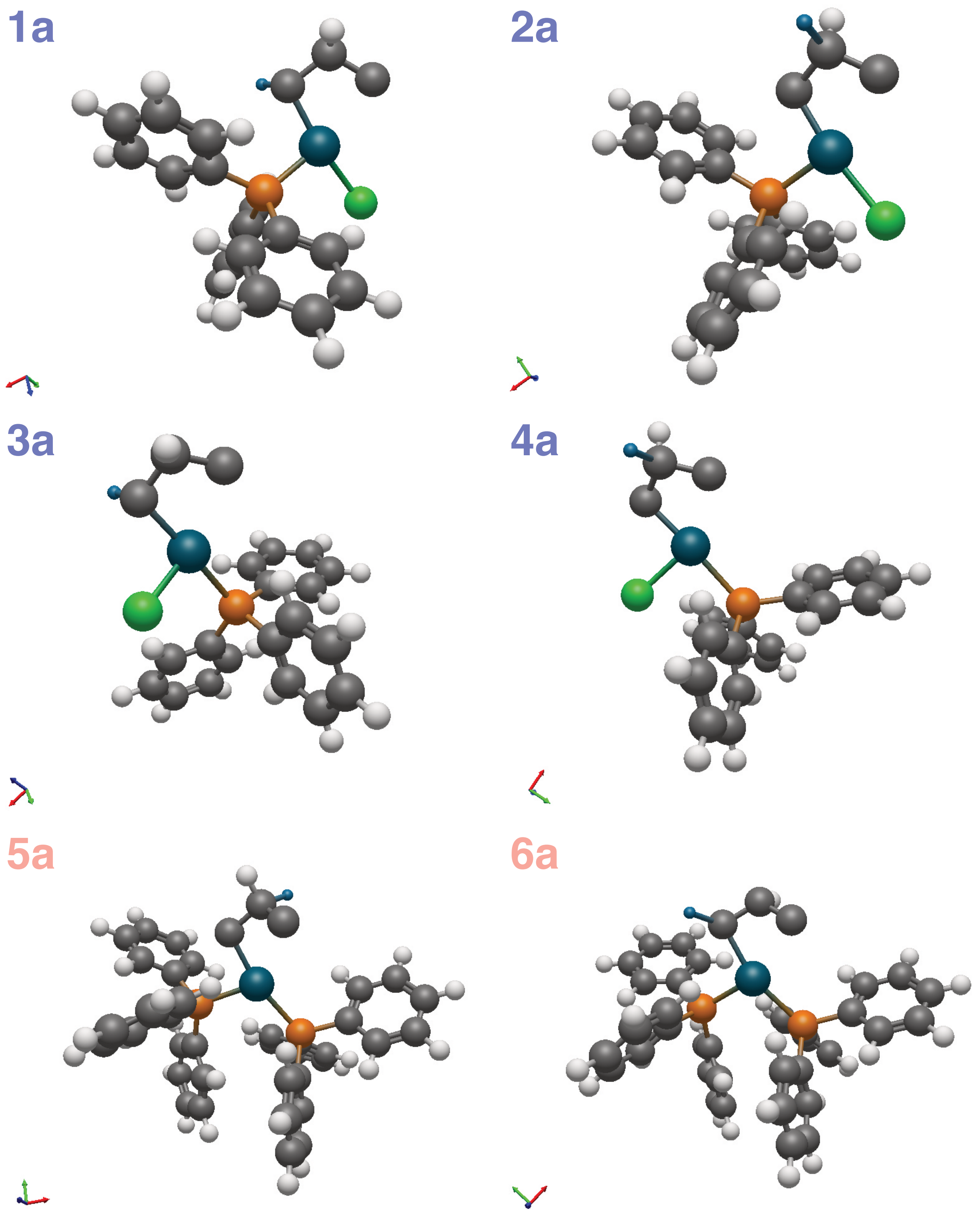

Figure S2: Graphical representations of the structural templates used to generate the post-insertion complexes. The blue dummy atom corresponds to the connecting R-group atom of the monosubstituted alkene, RC=C.

Table S1: Exp marked with * taken from ACR article, while other values taken from JOC 1992, 57, 3558. For the latter values the neutral results are for $(PPh_3)_2$ and X=I, while the cationic results are for X=triflate and DPPP. r^2SCAN-3c//xTB-GFN1 systematic random 50,000 conformers. "[]" is pure xTB-GFN1. The values in superscript are the relative energies in kJ/mol to the other regioisomer.

			Neutral		Cationic	
			Exp α/β	DFT (all)	Exp α/β	DFT (all)
a	O-n-butane	EDG	76/24	β^{23} [β^{54}]	>99/1	m [β^{25}]
b	2-pyrrolidone	EDG	64/36	m [β^{16}]	>99/1	α^{43} [α^{36}]
c	$N(CH_3)COCH_3$	EDG	71/29	β^{39} [β^{22}]	99/1	β^{18} [m]
g	OAc	EDG	65/35	β^{18} [m]	95/5	β^{11} [β^{38}]
i	N-succinimide	EDG	5/95	m [β^{16}]	62/38	α^{23} [α^{24}]
f	CH_2CH_2OH	EDG	20/80*	β^{26} [β^{14}]	90/10	α^{16} [m]
	n-butane	EDG	20/80*	β^{11} [m]	80/20*	m [β^{14}]
h	Ph	EWG	7/93	β^{46} [β^{20}]	38/62	β^{18} [β^{23}]
d	CH_2OH	EWG	0/100*	m [m]	>99/1	α^{17} [α^{26}]
e	$CH(OH)CH_3$	EWG	10/90*	m [β^{46}]	95/5	α^{24} [α^{27}]
k	Cyano	EWG	<1/99	β^{42} [β^{35}]	<1/99	β^{39} [β^{16}]
j	$COOCH_3$	EWG	<1/99	β^{15} [β^{25}]	<1/99	α^{18} [α^{32}]

Table S2: Exp marked with * taken from ACR article, while other values taken from JOC 1992, 57, 3558. For the latter values the neutral results are for $(PPh_3)_2$ and X=I, while the cationic results are for X=triflate and DPPP. r^2SCAN-3c//xTB-GFN1 CREST-GFNFF conformer search. "[]" is pure xTB-GFN1. The values in superscript are the relative energies in kJ/mol to the other regioisomer.

			Neutral		Cationic	
			Exp α/β	DFT (all)	Exp α/β	DFT (all)
a	O-n-butane	EDG	76/24	β^{22} [β^{25}]	>99/1	β^{58} [β^{55}]
b	2-pyrrolidone	EDG	64/36	β^{38} [β^{75}]	>99/1	m [α^{10}]
c	$N(CH_3)COCH_3$	EDG	71/29	m [β^{23}]	99/1	β^{12} [m]
g	OAc	EDG	65/35	β^{41} [β^{80}]	95/5	α^{32} [α^{33}]
i	N-succinimide	EDG	5/95	m [β^{43}]	62/38	α^{38} [α^{33}]
f	CH_2CH_2OH	EDG	20/80*	β^{14} [β^{64}]	90/10	α^{21} [m]
	n-butane	EDG	20/80*	m [m]	80/20*	α^{26} [α^{22}]
h	Ph	EWG	7/93	β^{44} [β^{34}]	38/62	m [β^{21}]
d	CH_2OH	EWG	0/100*	m [α^{27}]	>99/1	α^{38} [α^{25}]
e	$CH(OH)CH_3$	EWG	10/90*	α^{20} [α^{14}]	95/5	α^{39} [α^{17}]
k	Cyano	EWG	<1/99	β^{37} [β^{40}]	<1/99	β^{63} [β^{19}]
j	$COOCH_3$	EWG	<1/99	m [m]	<1/99	α^{23} [α^{19}]

A list of included precatalysts in the dataset:

– Reactions with monodentate ligands ($(PPh_3)_2$):

- tetrakis(triphenylphosphine) palladium(0)
- bis-triphenylphosphine-palladium(II) chloride/bromide
- palladium diacetate and triphenylphosphine
- palladium dichloride and triphenylphosphine
- bis-(dibenzylideneacetone)-palladium(0) and triphenylphosphine
- tris-(dibenzylideneacetone)dipalladium(0) and triphenylphosphine [Pd2dba3/PPh3]
- tris-(dibenzylideneacetone)dipalladium(0) chloroform complex and triphenylphosphine [Pd2(dba)3·CHCl3/PPh3]
- dichloro bis(acetonitrile) palladium(II) and triphenylphosphine

– Reactions with bidentate ligands (DPPP or DPPE):

- bis-(dibenzylideneacetone)-palladium(0) and dppe
- bis[1,2-bis(diphenylphosphino)ethane]palladium(0)
- palladium diacetate and dppe/dppp
- tris-(dibenzylideneacetone)dipalladium(0) and dppe/dppp

Reactions employing the following additives were excluded from the dataset:

['sec.-butyllithium', 'tert.-butyl lithium', 'lithium chloride', 'lithium carbonate']

A list of included additives in the dataset:

– Reactions with monodentate ligands ($(PPh_3)_2$):

['Dowex LX2-100 Bicarb form', 'potassium chloride', 'epichlorohydrin', 'N,N,N,N,-tetramethylethylenediamine', 'tetramethylammonium bromide', 'chloro[1,3-bis(2,4,6-trimethylphenyl)imidazol-2-ylidene]copper(I)', 'tetrabutylammomium bromide', 'trifluoroacetic acid', 'tetra-(n-butyl)ammonium iodide', 'piperidine', 'silver carbonate', '1-methyl-pyrrolidin-2-one', 'tributyl-amine', 'tri-tert-butyl phosphine', 'sodium acetate trihydrate', 'potassium acetate', 'hydrogenchloride', 'trimethylamine', "2'-(5H-benzo[b]phosphindol-5-yl)-[1,1'-biphenyl]-2-yl acetate", 'hydroquinone', 'styrene', 'potassium phosphate', '1-ethyl-piperidine', 'silver nitrate', '1,4-diaza-bicyclo[2.2.2]octane', '1-hexyl-3-methylimidazolium tetrafluoroborate', 'tetrabutyl-ammonium chloride', 'N-ethyl-N,N-diisopropylamine', '2,6-di-tert-butyl-4-methyl-phenol', 'water', 'tetraethylammonium chloride', 'acetic acid', 'anhydrous sodium carbonate', 'sodium iodide', 'potassium fluoride', '1-butyl-3-methylimidazolium Tetrafluoroborate', 'triethylamine', 'dicyclohexyl-carbodiimide', 'dicyclohexylmethylamine', 'sodium hydroxide', 'thallium(I) acetate', 'Dowex HC03-', 'formaldehyd', 'potassium carbonate', '3 A molecular sieve', 'dipotassium peroxodisulfate', 'anhydrous Sodium acetate', 'potassium hydroxide', 'sodium t-butanolate', 'lithium bromide', 'potassium hydrogencarbonate', 'norborn-2-ene', 'tetra-n-propylammonium bromide', 'sodium carbonate', 'sodium acetate', 'N,N-dimethyl-formamide', 'zinc dibromide', "(N,N'-phenylenebis(3,5-di-tert-butylsalicylideneiminato))zinc(II)", 'α,α,α-trifluorotoluene', 'cobalt(II) bromide', 'diisopropylamine', 'palladium(II) oxide', '1,3-bis-(diphenylphosphino)propane', 'sodium hydrogencarbonate', 'N-Methyldicyclohexylamine', 'copper dichloride', 'caesium carbonate', 'copper(l) iodide', '1,8-diazabicyclo[5.4.0]undec-7-ene', '1,5-diphenyl-1,4-pentadiene-3-one', '1-decyl-3-methyl-1H-imidazolium tetrafluoroborate', "dichloro(1,1'-bis(diphenylphosphanyl)ferrocene)palladium(II)*CH2Cl2", 'pyridine', 'N-benzyl-N,N,N-triethylammonium chloride', 'Dowex 1x2-100 Bicarb form', 'Tri(p-tolyl)phosphine', 'di-tert-butyl dicarbonate', 'silver(I) acetate', 'tetra(n-butyl)ammonium

hydrogensulfate', 'tris-(o-tolyl)phosphine', 'sodium bromide', 'C10H14O2']

– Reactions with bidentate ligands (DPPP or DPPE):

['hydrogenchloride', 'tetrafluoroboric acid', 'N,N-diisopropylammonium tetrafluoroborate', 'caesium carbonate', 'N-Bromosuccinimide', 'tetrabutylammomium bromide', 'sodium carbonate', 'sodium acetate', 'N-ethyl-N,N-diisopropylamine', 'tetrabutylammonium bromide', 'silver carbonate', 'diisopropylamine', 'tributyl-amine', 'triethylammonium tetrafluoroborate', 'sodium hydride', '1-butyl-3-methylimidazolium Tetrafluoroborate', 'triethylamine', 'thallium(I) acetate', 'potassium carbonate']

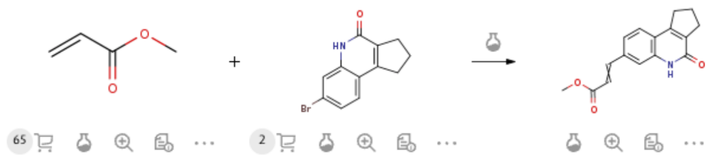

1 Conditions Find Similar Reaction ID: 22964783

Conditions	Yield	Reference
**With** triethylamine; tetrakis(triphenylphosphine) palladium$^{(0)}$ **In** DMF (N,N-dimethyl-formamide) at 120°C; for 3h;<br>Experimental Procedure		Current Patent Assignee: BAYER AG - EP1115708, 2004, B1<br>Location in patent: Page 10<br>KB links Details Abstract

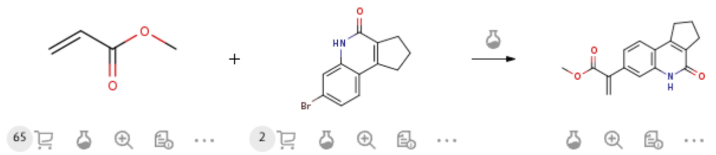

1 Conditions Find Similar Reaction ID: 24132152

Conditions	Yield	Reference
**With** triethylamine; tetrakis(triphenylphosphine) palladium$^{(0)}$ **In** N,N-dimethyl-formamide<br>Experimental Procedure	550 mg (99%)	Current Patent Assignee: BAYER AG - US6391887, 2002, B1<br>KB links Details Abstract

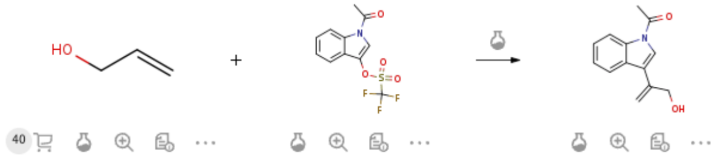

1 Conditions Find Similar Reaction ID: 5063282

Conditions	Yield	Reference
**With** triethylamine; palladium diacetate; triphenylphosphine **In** N,N-dimethyl-formamide at 100°C; for 18h; Substitution;	28%	Malapel-Andrieu, Beatrice; Merour, Jean-Yves<br>[**Tetrahedron**, **1998**, vol. 54, # 37, p. 11079 - 11094]<br>KB link Cited 40 times Details Abstract

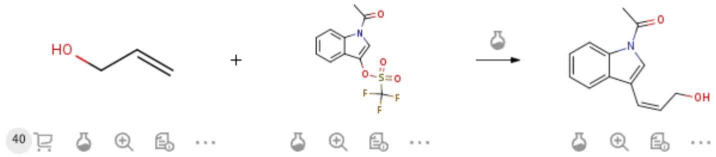

1 Conditions Find Similar Reaction ID: 4888015

Conditions	Yield	Reference
**With** palladium diacetate; triethylamine; triphenylphosphine **In** N,N-dimethyl-formamide at 100°C; for 5h;		Malapel-Andrieu, Beatrice; Merour, Jean-Yves<br>[**Tetrahedron Letters**, **1998**, vol. 39, # 1-2, p. 39 - 42]<br>KB link Cited 12 times Details Abstract

Figure S3: Example of identical reactions with different outcome.

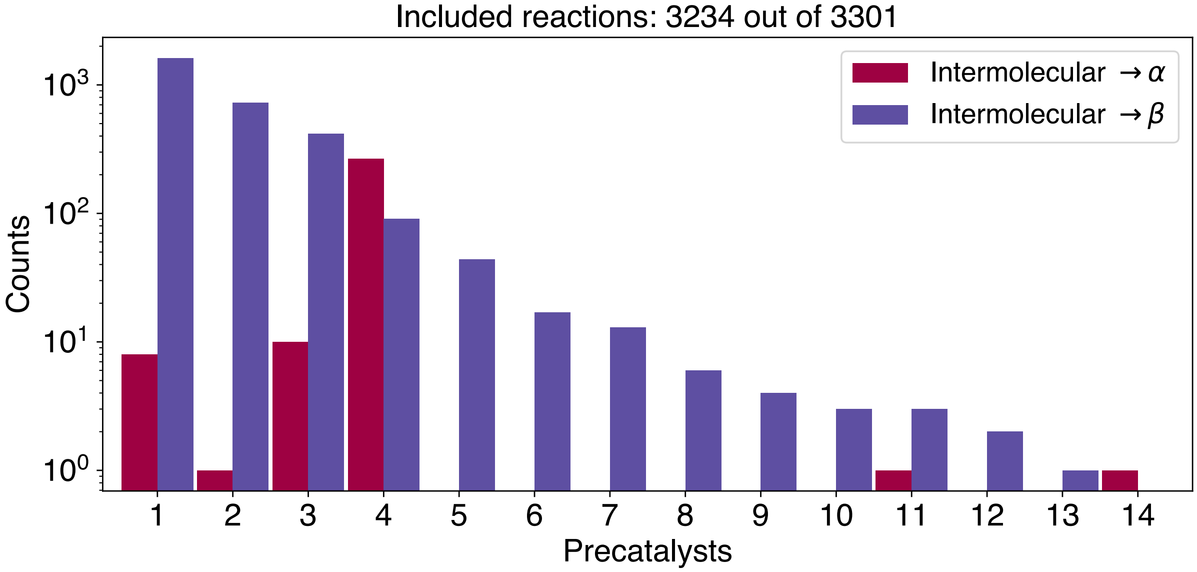

Figure S4: A bar plot of the reported precatalysts with respect to reactions employing DPPP, DPPE, or $(PPh_3)_2$ Pd-ligands. 1: 'Palladium diacetate and triphenylphosphine', 2: 'Bis-triphenylphosphine-palladium(II) chloride', 3: 'Tetrakis(triphenylphosphine) palladium(0)', 4: 'Palladium diacetate and dppp', 5: 'Palladium dichloride and triphenylphosphine', 6: 'Tris-(dibenzylideneacetone)dipalladium(0) and triphenylphosphine', 7: 'Palladium diacetate and dppe', 8: 'Tris-(dibenzylideneacetone)dipalladium(0) and dppe', 9: 'Bis-triphenylphosphine-palladium(II) bromide', 10: 'Bis-(dibenzylideneacetone)-palladium(0) and triphenylphosphine', 11: 'Tris-(dibenzylideneacetone)dipalladium(0) and dppp', 12: 'Bis-(dibenzylideneacetone)-palladium(0) and dppe', 13: 'Tris-(dibenzylideneacetone)dipalladium(0) chloroform complex and triphenylphosphine', 14: 'Bis[1,2-bis(diphenylphosphino)ethane]palladium(0)'.

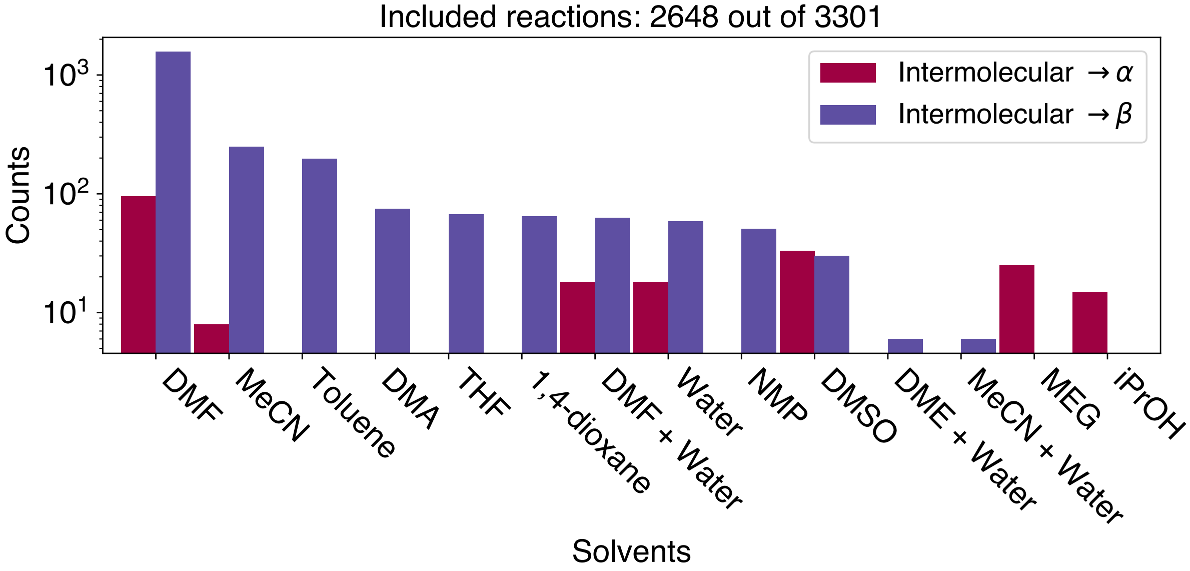

Figure S5: A bar plot of the reported solvents with respect to reactions employing DPPP, DPPE, or $(PPh_3)_2$ Pd-ligands. Solvents with less than 5 entries are not shown.

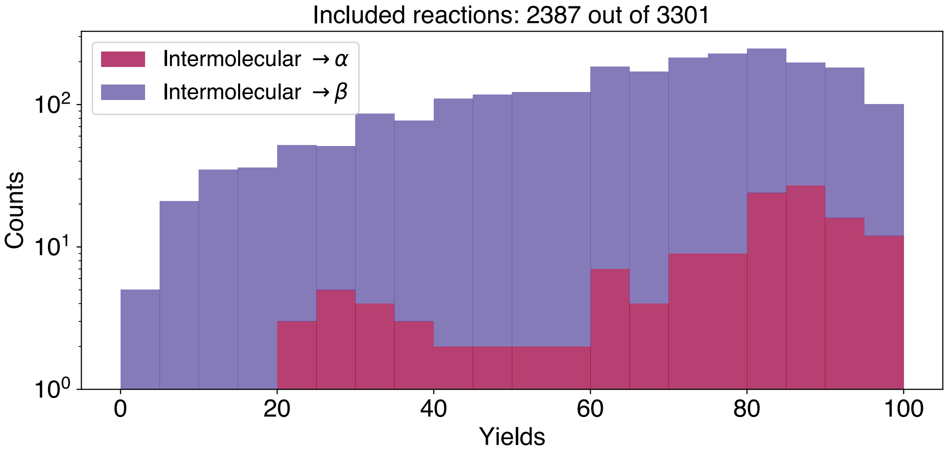

Figure S6: The distribution of reported yields with respect to reactions employing DPPP, DPPE, or $(PPh_3)_2$ Pd-ligands.

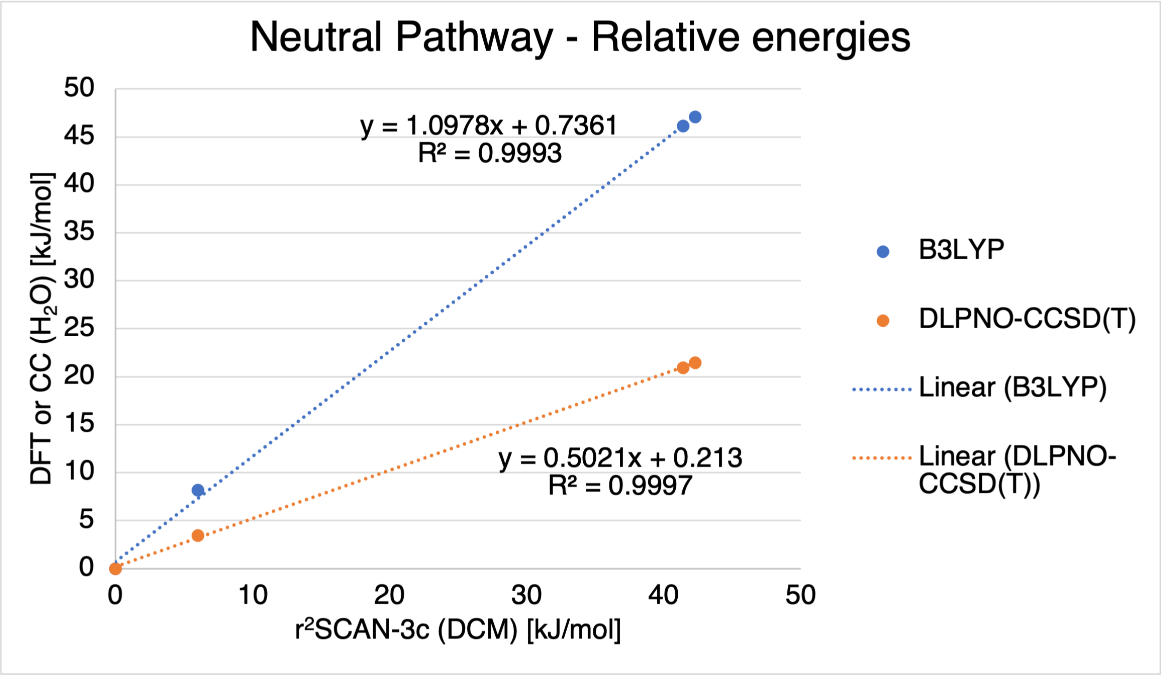

Figure S7: Correlation plot of single point B3LYP/def2-TZVP (H_2O) and DLPNO-CCSD(T)/def2-TZVPP (H_2O) energies vs. single point r^{2}SCAN-3c (DCM) energies for compound "a" in Table 1 w.r.t. the neutral pathway.

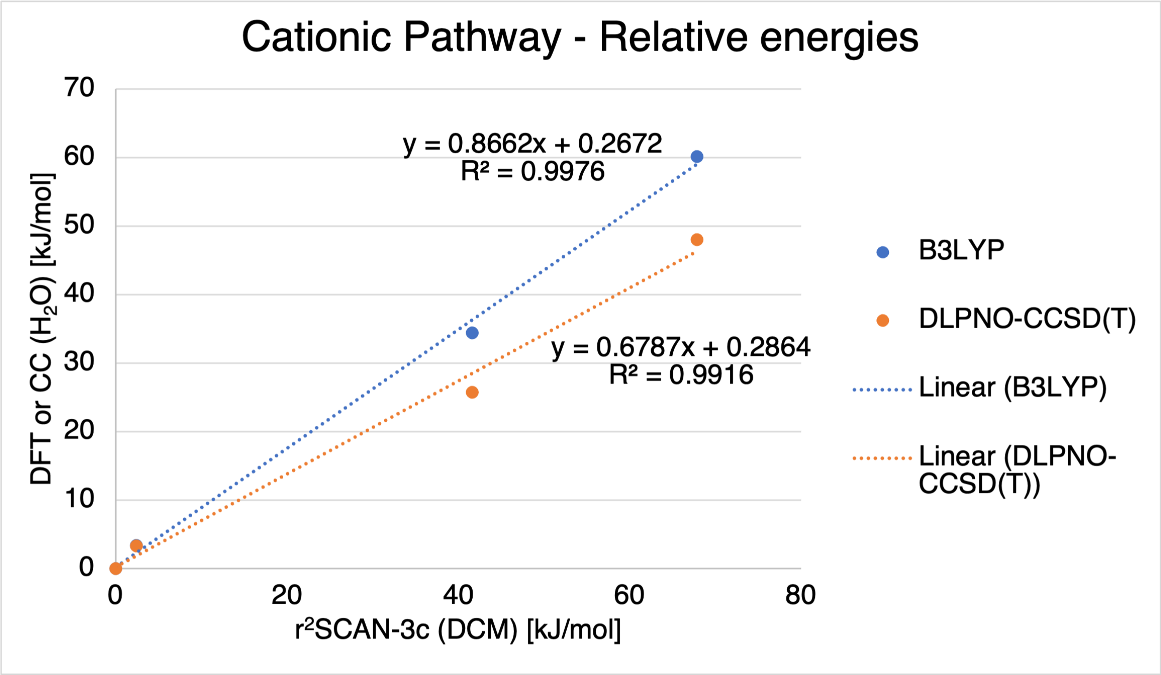

Figure S8: Correlation plot of single point B3LYP/def2-TZVP (H_2O) and DLPNO-CCSD(T)/def2-TZVPP (H_2O) energies vs. single point r^{2}SCAN-3c (DCM) energies for compound "a" in Table 1 w.r.t. the cationic pathway.

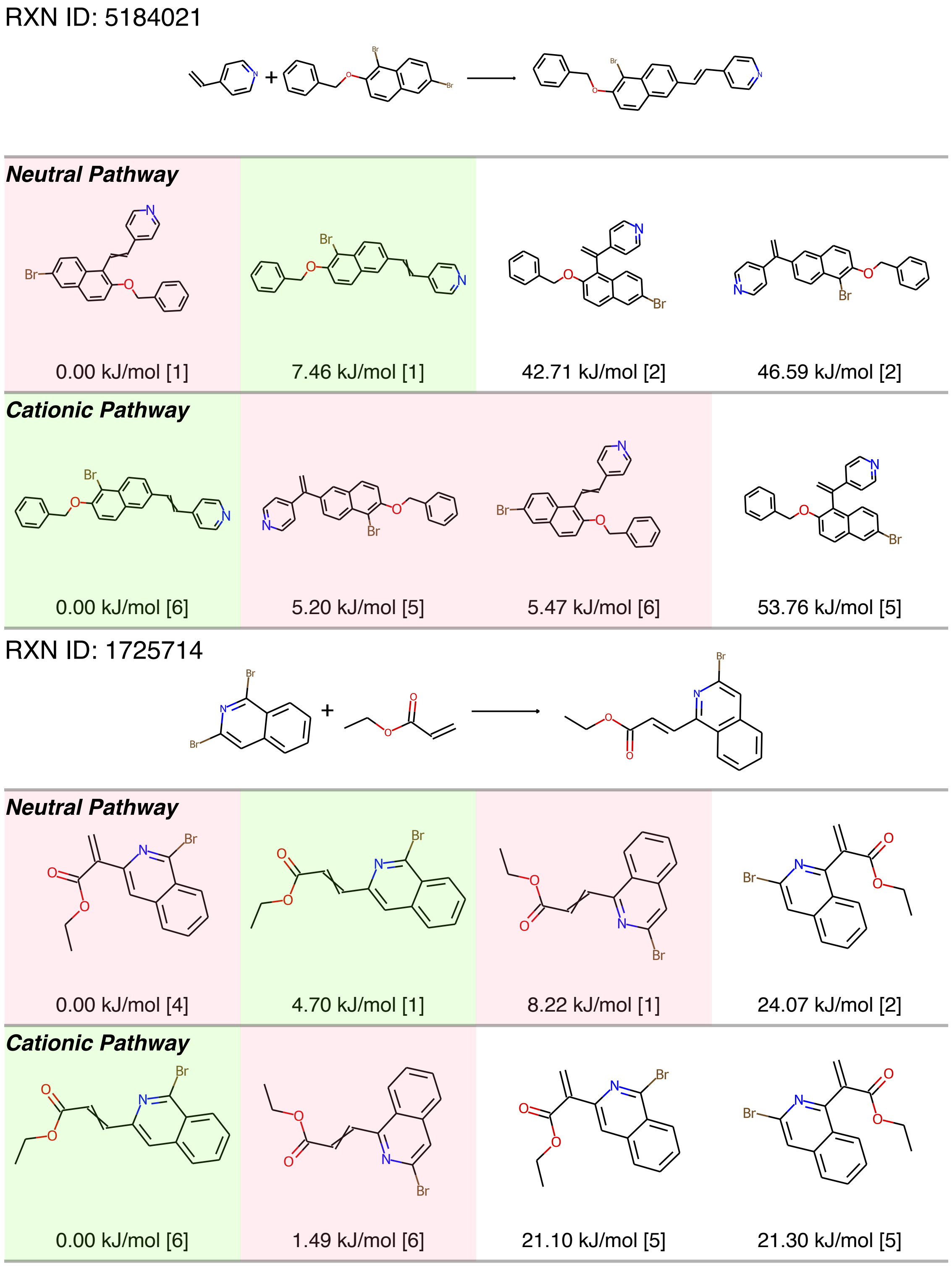

Figure S9: Reactions with multiple selectivity sites. The relative energies of the post-insertion complexes are obtained using r^{2}SCAN-3c//xTB-GFN2 level of theory. "Red" indicates predicted product(s) within the 3 kcal/mol ≈ 12.6 kJ/mol cutoff, "Green" indicates the reported product being among the predicted product(s), and "Grey" indicates that the reported product is not part of the predicted product(s).

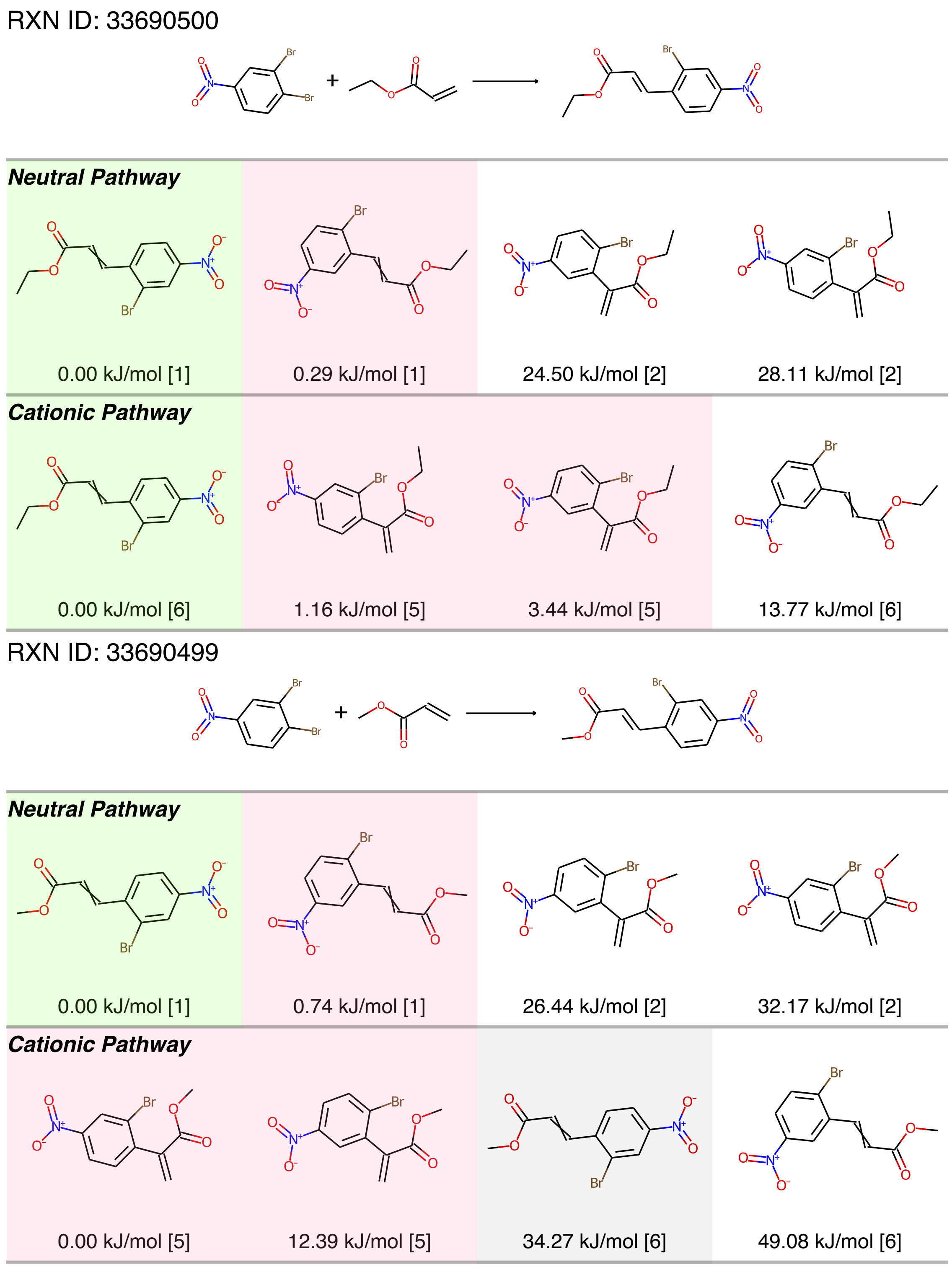

Figure S10: Reactions with multiple selectivity sites. The relative energies of the post-insertion complexes are obtained using r^{2}SCAN-3c//xTB-GFN2 level of theory. "Red" indicates predicted product(s) within the 3 kcal/mol ≈ 12.6 kJ/mol cutoff, "Green" indicates the reported product being among the predicted product(s), and "Grey" indicates that the reported product is not part of the predicted product(s).

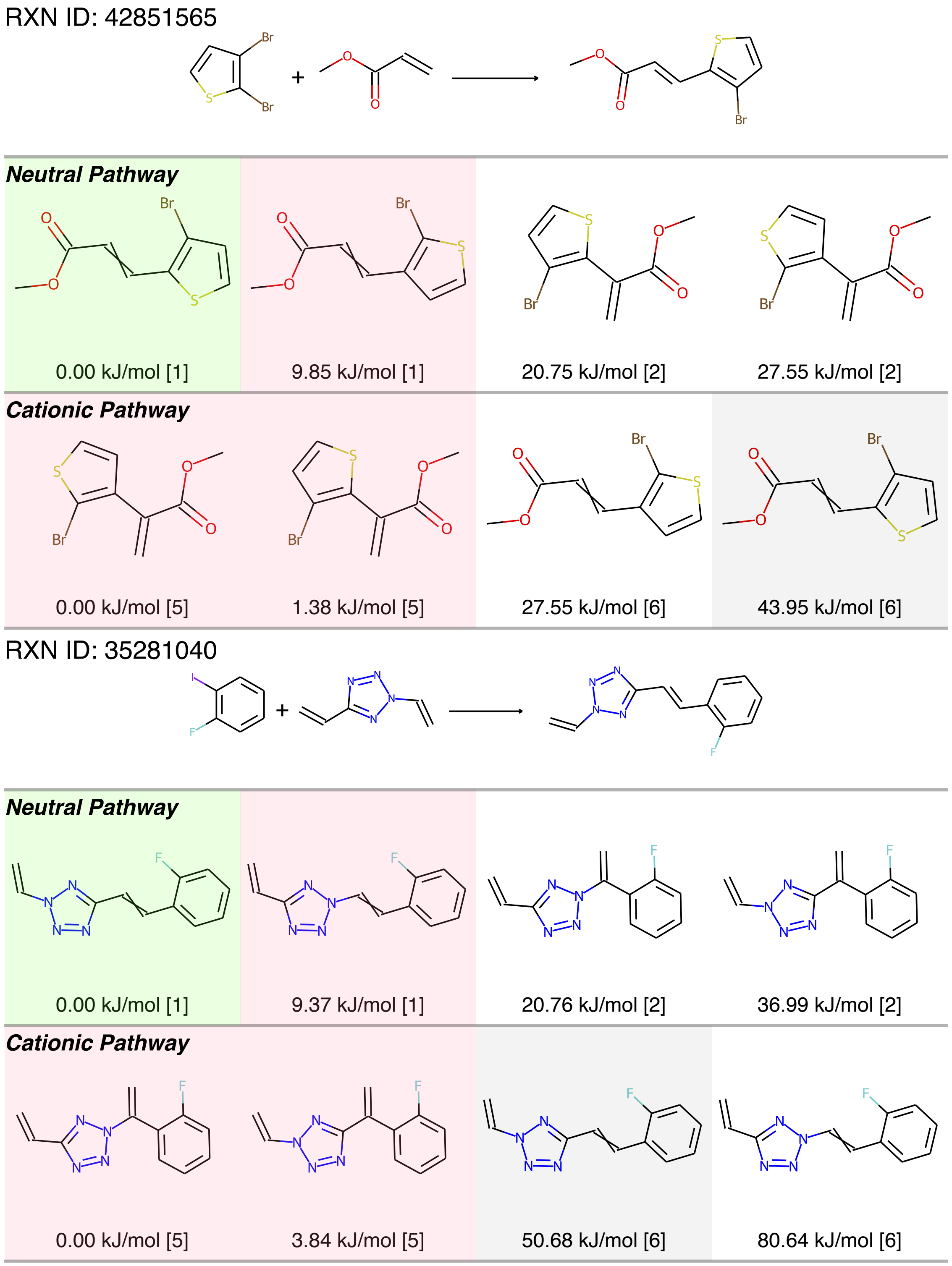

Figure S11: Reactions with multiple selectivity sites. The relative energies of the post-insertion complexes are obtained using r^{2}SCAN-3c//xTB-GFN2 level of theory. "Red" indicates predicted product(s) within the 3 kcal/mol ≈ 12.6 kJ/mol cutoff, "Green" indicates the reported product being among the predicted product(s), and "Grey" indicates that the reported product is not part of the predicted product(s).

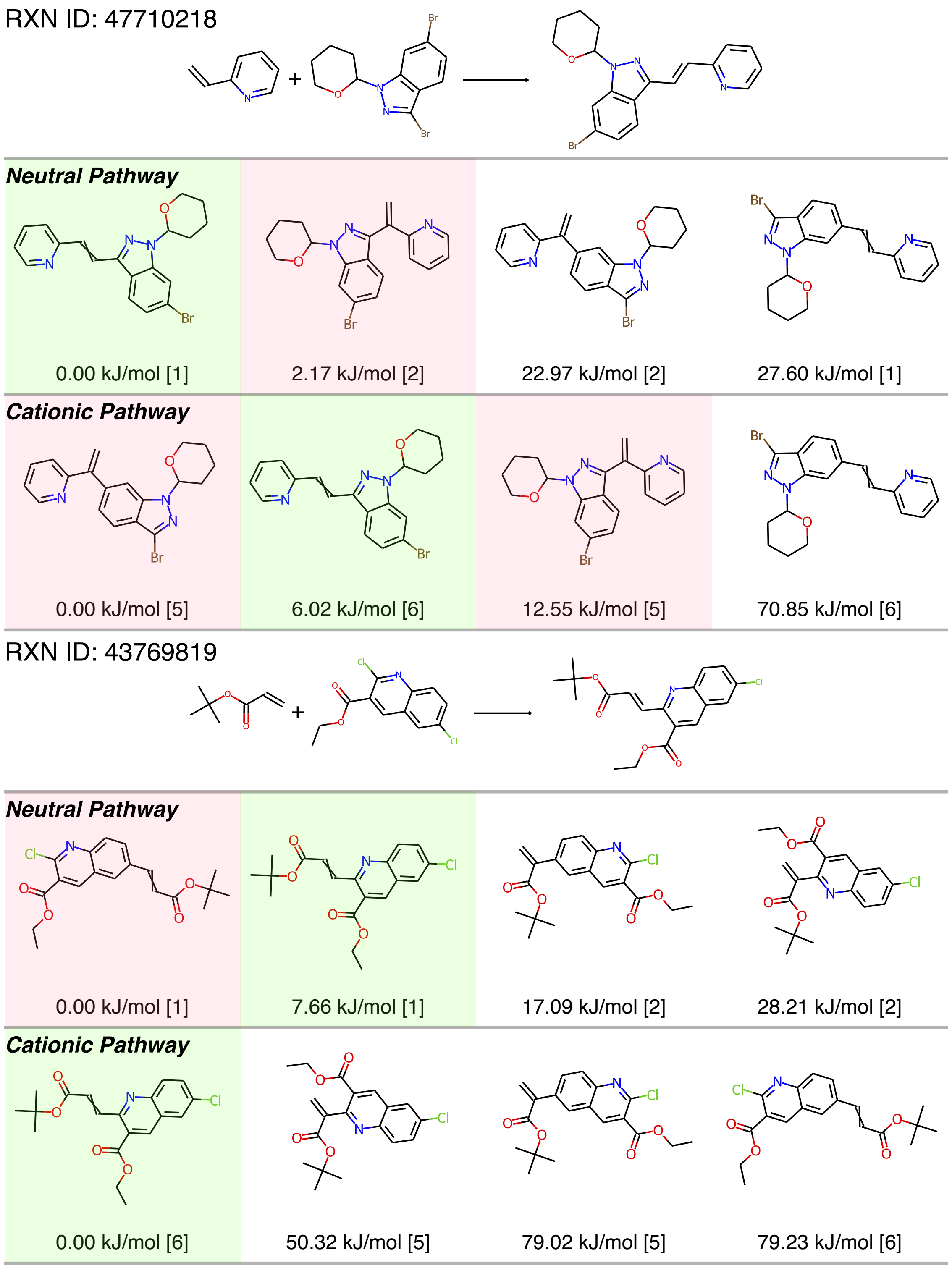

Figure S12: Reactions with multiple selectivity sites. The relative energies of the post-insertion complexes are obtained using r^{2}SCAN-3c//xTB-GFN2 level of theory. "Red" indicates predicted product(s) within the 3 kcal/mol ≈ 12.6 kJ/mol cutoff, "Green" indicates the reported product being among the predicted product(s), and "Grey" indicates that the reported product is not part of the predicted product(s).

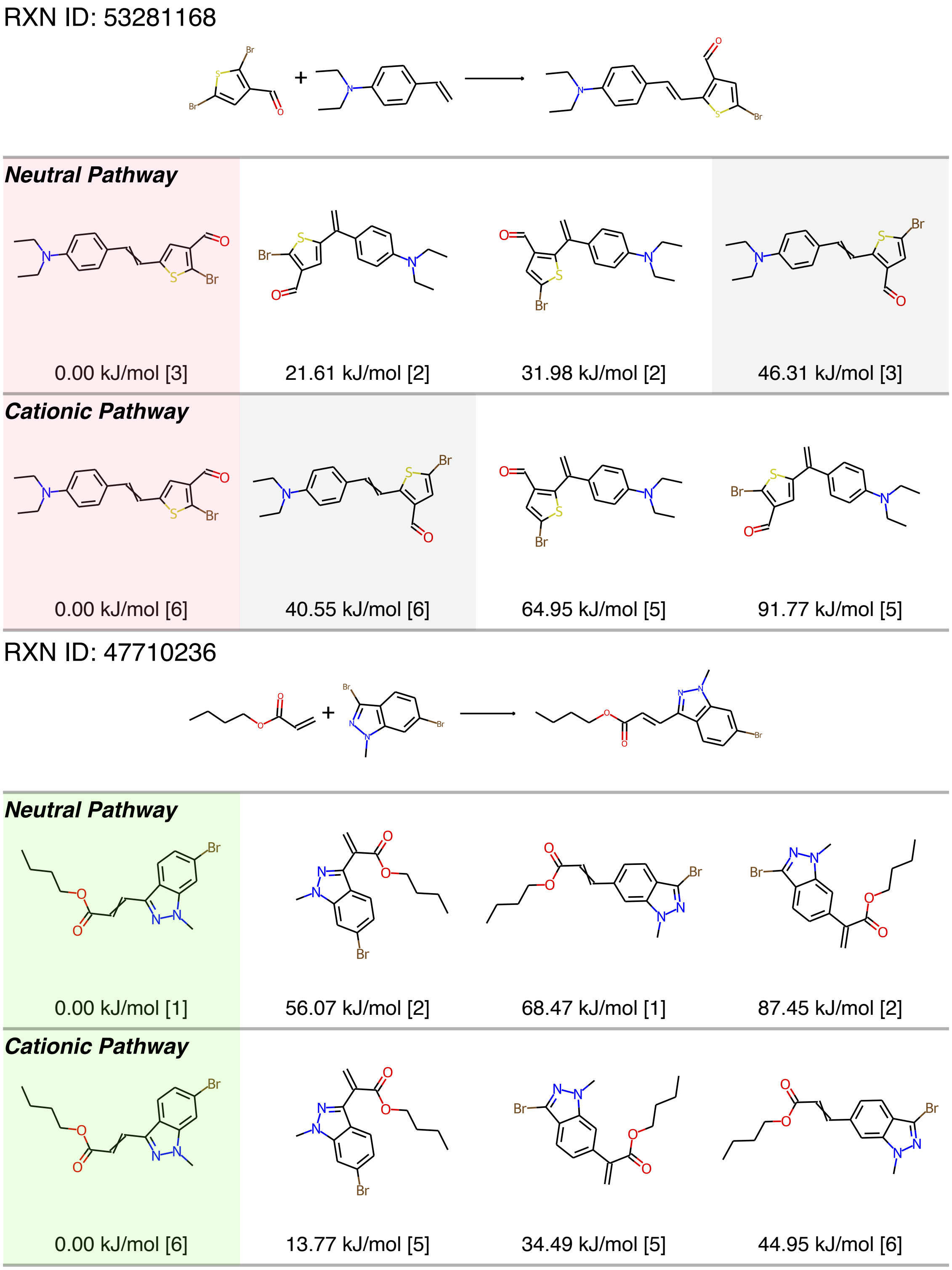

Figure S13: Reactions with multiple selectivity sites. The relative energies of the post-insertion complexes are obtained using r^{2}SCAN-3c//xTB-GFN2 level of theory. "Red" indicates predicted product(s) within the 3 kcal/mol ≈ 12.6 kJ/mol cutoff, "Green" indicates the reported product being among the predicted product(s), and "Grey" indicates that the reported product is not part of the predicted product(s).
